# New Hopes for Treatment of Alzheimer’s Disease

**Published:** 2018

**Authors:** Shahin Akhondzadeh

Alzheimer’s Disease (AD), the leading cause of dementia worldwide, is an irreversible progressive neurodegenerative disorder characterized by cognitive impairment and functional disability ^1,2^. Devastating nature of AD leads to serious social and economic impacts on the healthcare systems which implies the necessity of its proper management. It has been demonstrated that patients’ quality of life and their overall prognosis has a significant negative correlation with the severity of AD. Patients with severe AD need full-time care and assistance with some basic activities of daily living such as feeding and dressing in addition to severe deterioration in various domains of their cognitive functioning. Moreover, behavioral aberrancy and neuropsychiatric symptoms such as depression, apathy, psychosis, agitation, and aggression are observed more frequently in moderate to severe AD. Despite such an enormous burden, most practical guidelines focus on mild to moderate stages of the illness and there is still a serious lack of evidence regarding the management of severe AD. Among currently FDA-approved drugs, very few medications have shown to be effective in attenuating some of the AD-related symptoms in severe stages. Memantine, an N-methyl-D-aspartate (NMDA) receptor antagonist, and donepezil, an acetylcholinesterase inhibitor (ACEI) are the most widely accepted agents in this regard. Unfavorable side effects of these agents along with lack of optimal efficacy have led to many researches trying to find novel pharmacologic strategies for AD based on its underlying pathophysiological defects ^3^.

More than 400 clinical trials are currently looking at new treatments for AD and many of them are actively recruiting. Many of these studies are based on decreasing the harmful effects of a toxic protein called amyloid-beta in the brain, but others reflect a broadening range of possible treatment approaches based on other theories about AD.

For example, the importance of inflammation in exacerbation of amyloid-beta’s neuron-destroying effects has led to trials of medications with anti-inflammatory properties ^4^.

Serotonin neurotransmission failure is a demonstrated aspect of AD, and several experimental medications attempt to correct that problem. RVT-101 and LuAE58054 are two examples of medications that are in clinical trials. Altering the brain’s serotonin activity seems to help cognitive difficulties in schizophrenia, and may also prove helpful in cognitive difficulties associated with AD ^5^. In conclusion, a number of biotechnologists are working on effective drugs for treatment of AD and in particular severe type.
